# A meta-analytic review of the impact of ADHD medications on anxiety and depression in children and adolescents

**DOI:** 10.1007/s00787-022-02004-8

**Published:** 2022-05-26

**Authors:** Annie Bryant, Hope Schlesinger, Athina Sideri, Joni Holmes, Jan Buitelaar, Richard Meiser-Stedman

**Affiliations:** 1 Department of Clinical Psychology and Psychological Therapies, University of East Anglia, Norwich, UK; 2 Norfolk and Suffolk NHS Foundation Trust, Hellesdon Hospital, Drayton High Road, Norwich, UK; 3 MRC Cognition and Brain Sciences Unit, University of Cambridge, Cambridge, UK; 4 Radboud University, Houtlaan 4, 6525 XZ Nijmegen, Netherlands

**Keywords:** ADHD, Anxiety, Depression, Mental health, Children, Adolescents, Pharmacology, Randomised controlled trials, Side effects

## Abstract

Anxiety and depression are listed as common side effects for medications licensed for treating ADHD in children and adolescents. This meta-analytic review of randomised controlled trials aimed to explore the effect of medications on symptoms of anxiety and depression in children and adolescents with ADHD. A meta-analytic review of ADHD drug trials in children and adolescents was conducted. Random effects meta-analyses were conducted on anxiety and depression outcomes measured by validated psychological scales or side effect rating scales. Only 11% of eligible trials in this review reported anxiety and/or depression as an outcome or side effect, limiting the conclusions of the meta-analyses. Relative to placebo control, no significant effect of medication was found for symptoms of anxiety or depression in randomised controlled trials of ADHD medication in children and adolescents. This review highlights the systemic lack of mental health outcome reporting in child and adolescent ADHD drug trials. The importance of widespread implementation of standardised measurement of mental health outcomes in future trials is discussed.

**Supplementary Information:**

The online version contains supplementary material available at 10.1007/s00787-022-02004-8.

## Introduction

Attention-deficit hyperactivity disorder (ADHD) is a neurodevelopmental disorder characterised by persistent inattention and/or hyperactivity and impulsivity which affects approximately 5% of children worldwide [1–3]. Emotion dysregulation and irritability are associated with ADHD symptoms in childhood [4] and children with ADHD are more likely symptoms are more likely to develop an internalising disorder, such as depression or anxiety, than children without ADHD symptoms [5, 6]. Prognoses for children with both ADHD and an internalising disorder are worse than for those with either disorder alone. Comorbidity is associated with higher incidence of psychiatric hospitalisation, higher rates of suicide, poorer quality of life, poorer social functioning and poorer family functioning [7–11].

Reviews and meta-analyses have shown that methylphenidate, atomoxetine and other licensed medications are efficacious for reducing ADHD symptoms in children and young people e.g., [12, 13]. A practitioner review published by The European ADHD Guidelines Group (EAGG) reported these medications are generally well-tolerated but that adverse events (AEs) can occur [14]. AEs reported for ADHD drugs, with varying levels of frequency, include changes in cardiovascular symptoms, growth, mood, sleep, tics, seizures, suicidality and psychotic symptoms [13, 14]. A Cochrane review of randomised and non-randomised studies showed that methylphenidate use in children and adolescents may be associated with a high number of non-serious AEs, however the quality of the available evidence was low [15].

Internalising problems can arise in children taking medications for ADHD [16]. In the UK, child and adolescent drug safety information is published in the British National Formulary for Children (BNFC) including lists of side effects and their associated risk. For all the drugs currently licensed in the UK to treat ADHD in children and adolescents (methylphenidate, lisdexamfetamine, dexamfetamine, atomoxetine and guanfacine), the BNFC lists increase of anxiety and depression as common or very common side effects [17, 18]. Consistent with this, Tobaiqy and colleagues [19] found the most frequently reported drug-related side effects by parents of children taking ADHD medications in the UK were mood and emotional problems (28%). Similarly, a review of the US Food and Drug Administration AE reporting database for methylphenidate, atomoxetine, amphetamine and lisdexamfetamine found significant odds ratios for anxiety, depression, self-harm and suicidality in children and adolescents [20].

AEs are measured in various ways in child and adolescent drug trials, but there is currently no standardised method [21]. Some use drug-specific side effect rating scales (SERS) which list common side effects for a particular drug and ask the clinician and/or parent to rate the severity of the effect. For some AEs it can be appropriate to administer specific measures such as validated questionnaires or physical measurements. However, many drug trials rely solely on spontaneous reporting of AEs from children and/or parents. In the UK, once medications are licensed for use, monitoring of long-term AEs relies predominantly on spontaneous reporting schemes such as the Yellow Card Scheme (YCS). Post-licensing spontaneous reporting is limited which some argue raises serious safety concerns for child and adolescent patients on long-term medications [22]. Drug safety data that relies on spontaneous reporting is particularly concerning for AEs such as internalising problems (e.g., anxiety and depression), which may be less noticeable to parents and clinicians, and even young people themselves, compared to behavioural or physical changes.

Whilst the BNFC lists anxiety and depression as common side effects of licensed ADHD medications for children and adolescents, mental health outcomes are rarely measured or reported in ADHD drug trials and reviews of drug safety. For example, a large review of a decade of research on the safety of atomoxetine did not include anxiety or depression as an outcome [23]. Likewise, the NICE evidence report supporting guidelines on the pharmacological management of ADHD in children and young people did not feature depression or anxiety as outcome measures of interest [24]. However, both did include suicide as a key outcome reflecting that while anxiety and depression are rarely studied in ADHD drug research, suicide is more routinely considered.

There are only a few existing meta-analyses of mental health outcomes in randomised controlled trials for child and adolescent ADHD. Manos and colleagues [25] conducted a literature review of RCTs reporting emotional expression (EE) as an outcome of drug treatment for ADHD. Heterogeneity in the measurement and reporting of EE across studies limited the conclusions that could be drawn, leading the authors to recommend the use of standardised EE measurement guidelines for randomised controlled trials of ADHD medication in children. Coughlin et al. [26] found no significant difference between risk of anxiety in children taking stimulants between drug and placebo groups when a random effects model was used. Conversely, a meta-analysis of treatment emergent mood and emotion AEs by Pozzi et al. [27] found that anxiety was significantly reduced with methylphenidate treatment compared to placebo. Sadness was not significantly different between drug and placebo groups.

Previous meta-analyses rely on spontaneous reporting of AEs, and do not include data from validated psychological scales measuring mental health outcomes. In contrast, the present meta-analytic review of randomised controlled trials explores symptoms of anxiety and depression in children and adolescents taking ADHD medication by considering SERS and validated psychological measures of these constructs. The focus of this review is specifically on anxiety and depression, not other emotion or mood symptoms, due to the increased risk of children and adolescents with ADHD developing these disorders [28]. Furthermore, in line with recent evidence that ADHD may be better understood as a continuum of symptoms and associated burden [29, 30] inclusion criteria was not limited to participants with an ADHD diagnosis; participants with clinical levels of ADHD symptoms were also included. Understanding the impact of medications for ADHD on children’s internalising symptoms is crucial for informing clinical management of children’s ADHD and other potential comorbidities. Establishing what role, if any, medications play in the onset or maintenance of internalising problems of children with ADHD may contribute to understanding the relationships between depression, anxiety and ADHD symptoms in children and young people.

The current review aimed to answer two questions. First, what is the effect of taking ADHD medications compared to placebo on symptoms of anxiety in RCTs with children and young people? Second, what is the effect of taking ADHD medications compared to placebo on depressive symptoms in RCTs with children and young people?

## Methods

### Study protocol and search strategy

A systematic review was conducted following PRISMA guidelines [31] and the Cochrane Handbook for Systematic Reviews of Interventions [32]. A completed PRISMA checklist can be found in Supplementary Material Table 1. The study protocol was registered with PROSPERO on the 23rd September 2020 (CRD42020208755).

**Table 1:** Characteristics of the included studies

Study	Mean age (range) years	Comorbid inclusion criteria	Psychiatric and neurological exclusion criteria	Male %	MDD (%)	Anxiety Disorder (%)	Trial design	Drug	Dosage	Length of trial weeks	Com-parison groups	Outcome measures
									Mean or range per day			
Aman et al. [43]	8.8 (5–13)	ID	Motor handicap, ASD, psychotic symptoms, epilepsy, down syndrome	71.4	NR	NR	CO	MPH	0.4 mg/kg. Fixed	4	Fenfluramine and PLAC	RBPC -Anxiety/ Withdrawal scale
Bangs et al. [44]	14.4 (12–18)	MDD	In psychotherapy	73.2	100	NR	PG	ATX	1.2–1.8 mg/kg. Flexible	9	PLAC	CDRS-R
Brown and Sexson [45]	13.6 (12–14)	–	ID, gross neurological disorders	100.0	NR	NR	CO	MPH	0.15–0.5 mg/kg. Fixed	8	PLAC	CPRS—Anxiety subscale
Buitelaar et al. [46]	9.2 (6–13)	–	TD	93.8	15	42	CO	MPH	20 mg. Fixed	4	Pindolol and PLAC	BSSERS
Daviss et al. [47]	9.2 (7–12)	–	TD, MDD, PDD, ASD, ID, ED, psychosis	79.7	0	NR	PG	MPH	30.2 mg. Flexible	16	Clonidine and PLAC	PSERS
Dell’Agnello et al. [48]	9.8 (6–15)	ODD	ID, BD, psychosis, PDD, seizures, serious risk of suicide, drug/alcohol abuse, in psychotherapy	92.7	1.5	11	PG	ATX	1.2 mg/kg. Flexible	8	PLAC	CDRS-R
												SCARED
Geller et al. [49]	12.0 (8–17)	Anxiety disorder	PTSD, panic disorder, specific phobias, OCD, BD, psychosis, PDD, seizures, substance abuse, serious risk of suicide	64.8	4.5	100	PG	ATX	1.2 mg/kg. Flexible	10	PLAC	MASC
Greenhill et al. [50]	9.0 (6–16)	–	Any psychiatric diagnosis, seizure, TD, ID	80.1	NR	NR	PG	MPH	40.7 mg. Flexible	3	PLAC	PSERS
Griffiths et al. [51]	11.29 (6–17)	–	Any psychotic or neurologic condition, alcohol, nicotine or drug use	78.5	2.6	38	CO	ATX	1.35 mg/kg. Flexible	6	PLAC	STAI and STAI-C
												DASS
Kurowski et al. [52]	11.5 (6–17)	TBI	Preinjury diagnoses of developmental or neurological disorders, psychiatric inpatient in past 12 months	76.9	NR	NR	CO	MPH	18–54 mg. Flexible	4	PLAC	PSERS
Lin et al. [53]	10.92 (6–17)	–	BD, psychosis, seizure, PDD, TD, anxiety	70.1	0.9	0.9	PG	MPH	18–54 mg. Fixed	8	Edivoxetine and PLAC	CBRS
Michelson et al. [54]	11 (8–18)	–	ID, psychosis or BD, seizure disorder, ongoing use of psychoactive drugs	71.6	0.6	0.6	PG	ATX	0.5–1.8 mg/kg. Fixed	8	PLAC	CDRS-R
Pliszka et al. [55]	7.95 (6–11)	–	MDD, depressed mood, manic episode, TD, psychosis or psychotic symptoms	NR	0	16	PG	MPH	25–50 mg. Flexible	3	Adderrall (mixed amphetamines) and PLAC	MTA-SERS
Ramtvedt et al. [56]	11.3 (9–14)	–	ID, psychosis, TBI, epilepsy, sensory deficits and/or motor impairment	79.4	NR	NR	CO	MPH	40 mg. Fixed	2	Dextroamphetamine and PLAC	BSSERS

*NR* not reported, *ASD* autism spectrum disorder, *BD* bipolar disorder, *ED* eating disorder, *ID* intellectual disability, *MDD* major depressive disorder, *OCD* obsessive compulsive disorder, *ODD* oppositional defiant disorder, *PDD* pervasive developmental disorder, *PTSD* post traumatic stress disorder, *TBI* traumatic brain injury, *TD* tic disorder, *ATX* atomoxetine, *MPH* methylphenidate, *BSSERS* barkley stimulant side effect rating scale, *CBRS* conners comprehensive behaviour rating scale, *CDRS-R *children’s depression rating scale revised, *CPRS* conners parent rating scale, *DASS* depression, anxiety and stress scale, *MASC* multidimensional anxiety scale for children, *MTA-SERS* multi-modality treatment of ADHD side effects scale, *PSERS*  pittsburgh side effect rating scale, *RBPC* revised behaviour problem checklist, *SCARED* screen for child anxiety related emotional disorders, *STAI* state and trait anxiety index, *STAI-C *state and trait anxiety index for children

Three electronic databases, PubMed, EMBASE and PsycINFO, were searched from the earliest publication date up to 13th November 2020. The search terms were: *attention deficit hyperactivity disorder* or *ADHD* or *ADD* or *hyperkinetic* or *hyperkinesis* AND *Amphetamine* or *amfetamine* or *methylphenidate* or *guanfacine* or *atomoxetine* or *clonidine* or *dexamphetamine* or *dexamfetamine* or *lisdexamfetamine* or *Ritalin* AND Child* or adolesc* or *paediatric* or *pediatric* AND *randomised controlled trial* or *randomized controlled trial* or *RCT.* Where appropriate, searches were also run using medical search headings (MeSH terms) or subject headings for ADHD and results combined with those using ADHD terms listed above. Terms were searched in titles and abstracts except, where possible, the RCT terms were searched in publication type. Filters were: English language and human studies.

### Study selection

Titles and abstracts were reviewed by the principal investigator to remove studies which clearly met exclusion criteria. The resulting shortlist of potentially eligible trials were retrieved in full text to determine whether they satisfied the inclusion and exclusion criteria. An independent researcher (trainee clinical psychologist) reviewed a randomly selected 20% of the full text articles (*n* = 43) to provide additional checking in line with the criteria. There were no disagreements on trial eligibility between the principal investigator and independent researcher.

The inclusion and exclusion criteria were as follows. The population of interest was children and adolescents aged 5–18 years old. To be included the studied populations must have met criteria for ADHD/ADD/hyperkinetic disorder or a similar term according to the Diagnostic and Statistical Manual of Mental Disorders (DSM) or the International Statistical Classification of Diseases and Related Health Problems (ICD), or had clinical levels of ADHD symptoms according to validated rating scales. If these conditions were met the populations were included regardless of ADHD subtype/presentation, gender, IQ and psychiatric or neurological comorbidities.

The included interventions were UK licensed pharmacological stimulant or non-stimulant treatments for ADHD. The included drug types were those that feature in NICE guidance on management of ADHD in children and adolescents [19]: methylphenidate, lisdexamfetamine, dexamfetamine, atomoxetine, guanfacine. Typical alternative spellings and drug names for these medications were included in the search terms if they returned additional results, as listed above.

The outcome of interest was ratings of depression or anxiety before and following a child or adolescent taking medication for ADHD. Ratings of depression and/or anxiety were extracted from SERS and/or standardised, validated psychological scales measures of depression and/or anxiety in children and adolescents, including parent, teacher, clinician and self-report measures.

The trials included were randomised placebo-controlled trials; both parallel-group and crossover trials. Aligned with the approach of the NICE guidance evidence review, the medication and placebo arms must have been administered for at least 2 weeks for a trial to be included (i.e., trials of short term or single dose effects were excluded) [24]. Trials which used a decreasing or discontinued medication dose (withdrawal or discontinuation studies) were excluded. Trials without a solely placebo drug arm (i.e. trials which administered a placebo drug in addition to another intervention such as psychotherapy) were excluded.

## Data extraction and risk of bias

### Data extraction

Data were extracted from the trials that fit the inclusion criteria. Where trials reported use of a SERS or validated psychological measure of anxiety and/or depression before and after medication, but did not report scores, trial authors were contacted to request the data. For included trials, data were extracted and tabulated in a unique data extraction form. Missing data were imputed in line with the Cochrane handbook [33]. Participant demographic information and details about the intervention and placebo conditions were extracted in addition to primary outcomes of depression and/or anxiety. Descriptive and outcome data were entered into Review Manager (RevMan) version 5.4 for systematic analysis [34].

### Data synthesis

#### Change from baseline vs. post-treatment outcomes

All available outcome data (both change from baseline and/or post-treatment outcome) were extracted from included studies.

#### Multiple intervention arms

Data from trials involving multiple intervention arms were handled as recommended in the Cochrane Handbook [35].

#### Crossover trials

Where appropriate, crossover trials were included in meta-analyses alongside parallel-group trials because both can be analysed together in a meta-analysis when they are used to study the same treatment effect [35, 36].

#### Multiple outcome measures

Multiple reporters provided outcome data across the different trials. For this reason, a hierarchy of preferred reporter was determined for data extraction as follows, beginning with first preference: child self-report, parent-report, clinician-report and teacher-report [37, 38]. Where trials reported multiple outcome measures with different reporters, the choice of measures included in the meta-analyses was based on the reporter hierarchy.

For trials with multiple outcome measures with the same reporter, the psychometric properties of the outcome measure influenced data extraction choice. Validated, standardised scales designed to measure the presentation of anxiety and/or depression were favoured over scales designed to measure a different presentation with the inclusion of a subscale tapping anxious or depressive symptoms.

### Data analysis

#### Data extraction and computation for analysis

For the validated measures, post-treatment *n*, mean and standard deviation (SD) for the drug and placebo group were extracted and entered into RevMan as continuous outcomes. For the SERS, n and percentage of children with the presence of anxiety or depression as a side effect as rated on the target item for both the drug and placebo group were extracted. SERS data were entered into RevMan using the generic inverse variance method.

#### Analysis plan

To allow for heterogeneity, random-effects meta-analysis was used [39] and the *l*^2^ statistic [40] was used to assess heterogeneity of effect sizes. RevMan was used to conduct the statistical analysis.

Four meta-analyses were conducted: two for anxiety outcomes and two for depression outcomes. Separate meta-analyses were conducted on data from validated measures and data from SERS items, for both anxiety and depression. The validated measures and SERS data were meta-analysed separately to reduce heterogeneity. The outcomes from the two measurement approaches were deemed too qualitatively different to justify analysing them together (i.e., a validated and reliable multi-item measure of anxiety holds greater qualitative weight compared to a single Likert-rated anxiety item on a side effect scale when interpreting information about a child’s mental health).

For the meta-analyses of validated measures, effect sizes for medication relative to placebo (based on post-treatment or change scores) were calculated. The included trials used different outcome measures, so the standardised mean difference (SMD) was used as the summary statistic. For the meta-analyses on SERS data, log odds ratios and their standard errors were calculated. Overall odds of having a side effect of depression or anxiety (indicated by an item score) were compared between drug and placebo groups.

#### Risk of bias analysis

The principal investigator used the Cochrane revised tool for risk of bias in randomised trials (RoB 2) [41] to assess the quality of the included trials. Versions of RoB 2 for individually randomised trials and crossover trials were used as appropriate for each trial. The effect of interest was adherence to the intervention. An independent researcher (a graduate-level assistant psychologist) was trained in using the RoB 2 tools and carried out independent assessments of risk of bias for the included trials. There were no disagreements on risk of bias assessments between the principal investigator and independent researcher.

The RoB 2 [41] was used to assess the quality of the included parallel group trials, and the additional guidance was followed for assessing quality of the crossover trials [42]. When completing the RoB 2 analysis, it was held in mind that the outcome of interest in the present review (anxiety and depression) was not the primary outcome in many of the included trials so bias ratings were considered as appropriate to the original design and aim of each trial.

#### Sensitivity analyses

Sensitivity analyses were conducted for meta-analyses that included both trials with change scores and trials with post-treatment scores, to test whether the overall effect size was robust to the origin of the SMD.

For trials that reported a SERS, some trials reported the presence of a side effect as represented by any score on the anxiety and/or depression items and some reported the presence of at least moderate scores on anxiety and/or depression items. Sensitivity analyses including only trials reporting at least moderate anxiety and/or depression item scores were conducted to test whether the meta-analyses effects were robust to the rated severity of the anxiety and/or depression side effect.

## Results

### Included studies

#### Search results

**Fig. 1:**
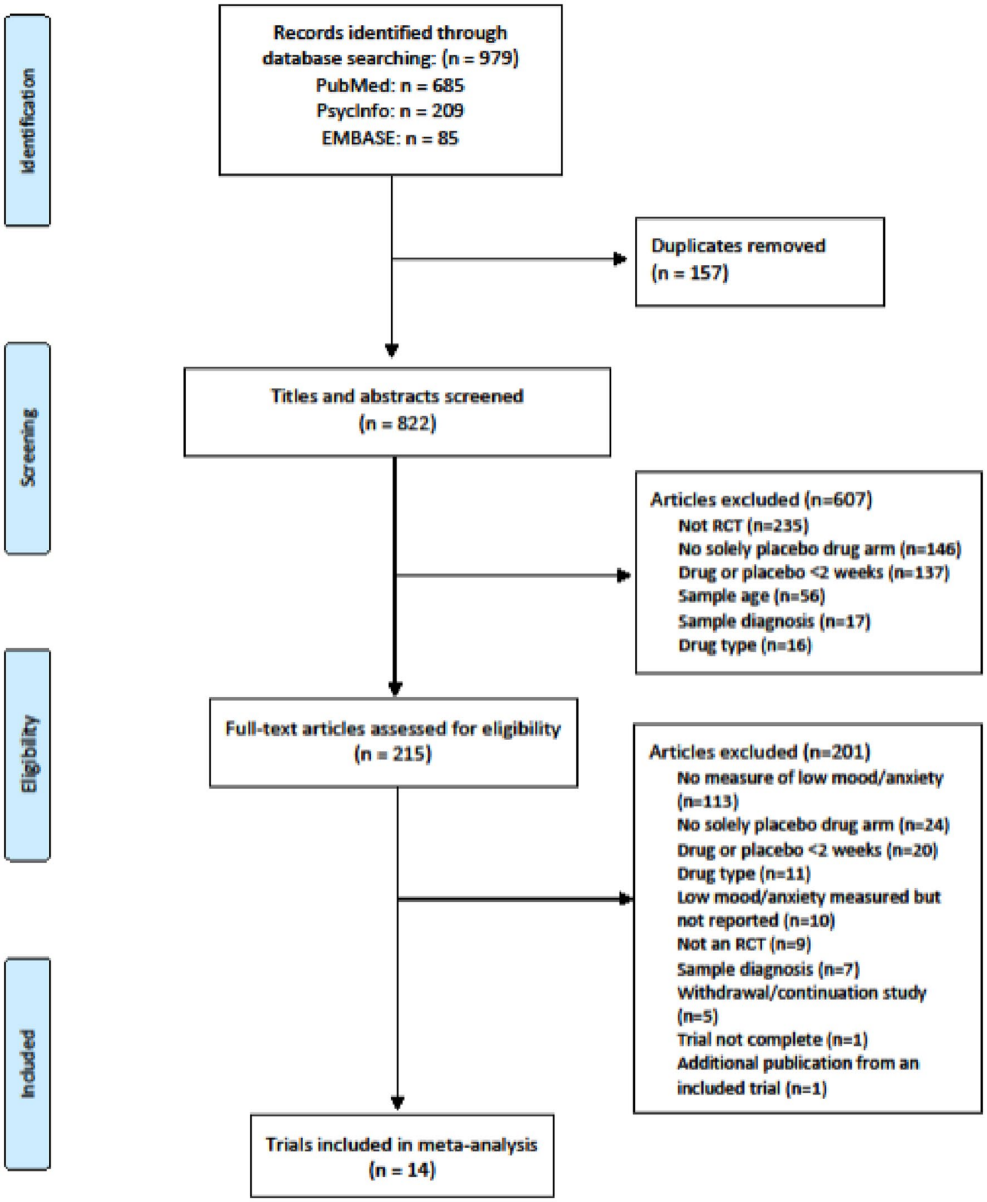
PRISMA flowchart for the systematic review

Figure 1 presents a PRISMA flowchart [31] of the study selection and exclusion process. The electronic database searches identified 979 citations. After collation and removal of duplicates, 822 articles were screened by title and abstract. A total of 215 full-text articles were assessed for eligibility resulting in 14 randomised controlled trials being selected for inclusion in the review [43–56]. The most common cause for exclusion (113 trials) was a lack of a reported measure of mood and/or anxiety.

#### Characteristics of included studies

Table 1 presents the characteristics of the included studies and baseline demographics of the included participants. Sample sizes of complete outcome data ranged from 22 [45] to 316 [50]. Ages of participants ranged from 5 to 18 years with a combined mean age of 10 years 8 months. Across the available information, 76.6% of participants were male and 78.4% were Caucasian. All included trials confirmed diagnoses of ADHD/ADD according to DSM criteria (DSM version appropriate to time of trial). There was insufficient data to report collectively on participant’s previous medication use or on trial discontinuation. Exclusion criteria in all trials involved some psychiatric and/or neurological disorders or symptoms. Four trials excluded young people with anxiety and/or depression from trial entry. Five trials recruited participants with a comorbid condition alongside ADHD.

The active treatment medication in nine trials was methylphenidate (mean daily dose 20–54 mg), while for the other five, it was atomoxetine (mean daily dose 0.5–1.8 mg/kg). The combined mean duration of trial arms was 7 weeks. Eight trials compared the active treatment medication directly with a placebo arm. Six trials also included another active medication arm, outcome data for which were not included in this meta-analysis.

Of the 215 full text articles assessed for eligibility, 10 trials reported having used a measure of anxiety and/or depression but did not report any data. Trial authors were contacted via email but no further data were received. For the 14 trials included in this review that did report outcome data, anxiety and/or depression was measured using validated questionnaire scales in eight trials and using SERS in six trials. Information on the included outcome measures is presented in Supplementary Material.

#### Risk of bias

**Fig. 2:**
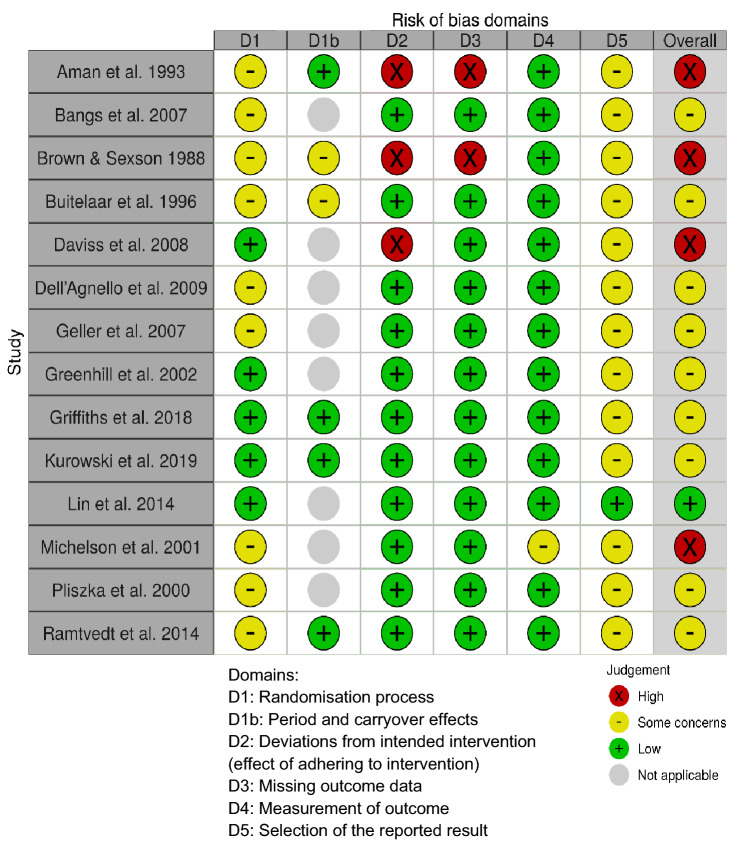
Risk of bias analysis plot of included studies

Figure 2 presents the risk of bias plot for the included studies, created using the robvis tool [57]. The plot was edited to reflect the additional domain (Domain S) included for crossover trials [42].

A lack of detailed information about randomisation processes raised concerns about risk of bias from prognostic factors that could predict the outcome by influencing allocation to intervention groups. The absence of information on intervention adherence in a handful of trials resulted in high risk of bias from deviations from intended interventions and risk of bias from missing outcome data. However, the majority of trials were rated as low risk of bias for these two domains. The absence of pre-specified analysis plans for most trials resulted in some concerns of a risk of bias from the selection of the reported result. There was an overall low risk of bias both in the measurement of outcomes and from period or carryover effects. Overall, the included studies showed at least some concerns, if not high risk of bias, across the described domains. The effect estimates included in the meta-analyses are at notable risk of being biased.

### Meta-analyses of effects on anxiety and depression: ADHD drugs vs. placebo

#### Validated questionnaire measures data

**Fig. 3:**
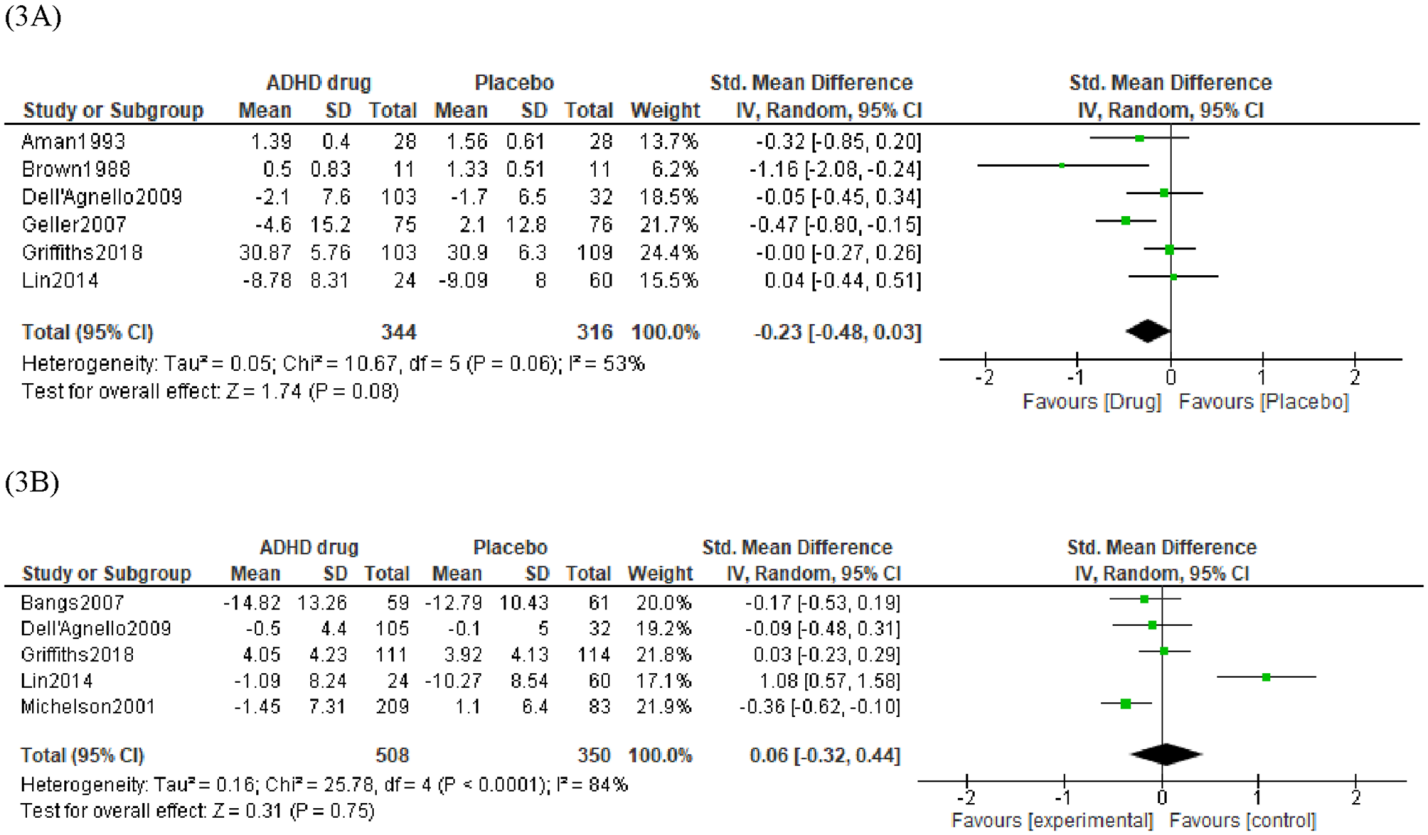
Forest plot of comparison between ADHD drug group and placebo group on Anxiety **A** and Depression **B** as measured by validated questionnaires

Cohen’s [58] effect sizes were used to interpret the SMD: 0.2 a small effect, 0.5 a moderate effect and 0.8 a large effect. As measured by validated questionnaires, anxiety was lower for children receiving ADHD medication over placebo, however, the magnitude of the effect was small and non-significant, (SMD = − 0.23, 95% CI = − 0.48 to 0.03, *p* = 0.06, *n* = 660, *k* = 6). The proportion of heterogeneity effects was modest (*I*^2^ = 53%). Visual inspection of the forest plot in (Fig. 3A) identified one trial, Brown and Sexson [45], as an outlier. This trial was identified as having a high risk of bias. A sensitivity analysis excluding this trial resulted in a smaller, and again non-significant, effect size (SMD = − 0.16, 95% CI = − 0.37 to 0.05 [favouring ADHD medication], *p* = 0.18, *n* = 638, *k* = 5).

For depression (see Fig. 3B) measured by validated questionnaires, the magnitude of the effect was negligible and non-significant (SMD = 0.06 [lower for placebo], 95% CI = − 0.32 to 0.44, *p* = 0.75, *n* = 858, *k* = 5). A substantial level of heterogeneity was indicated (*I*^2^ = 84%).Visual inspection of the forest plot in (Fig. 3B) identified one trial, Lin et al. [53], as an outlier as there was a much larger improvement in depression scores in the placebo group than in the drug group. The only clear difference between this trial and the others in the analysis was that Lin et al. [53] was a trial of methylphenidate vs. placebo, whereas the other trials all used atomoxetine vs. placebo. A sensitivity analysis excluding this trial resulted in an increased, but still small and non-significant, effect size (SMD = − 0.15, 95% CI = − 0.34 to 0.04, *p* = 0.11, *n* = 774, *k* = 4) where depression was lower for ADHD drugs over placebo.

### SERS item data

**Fig. 4:**
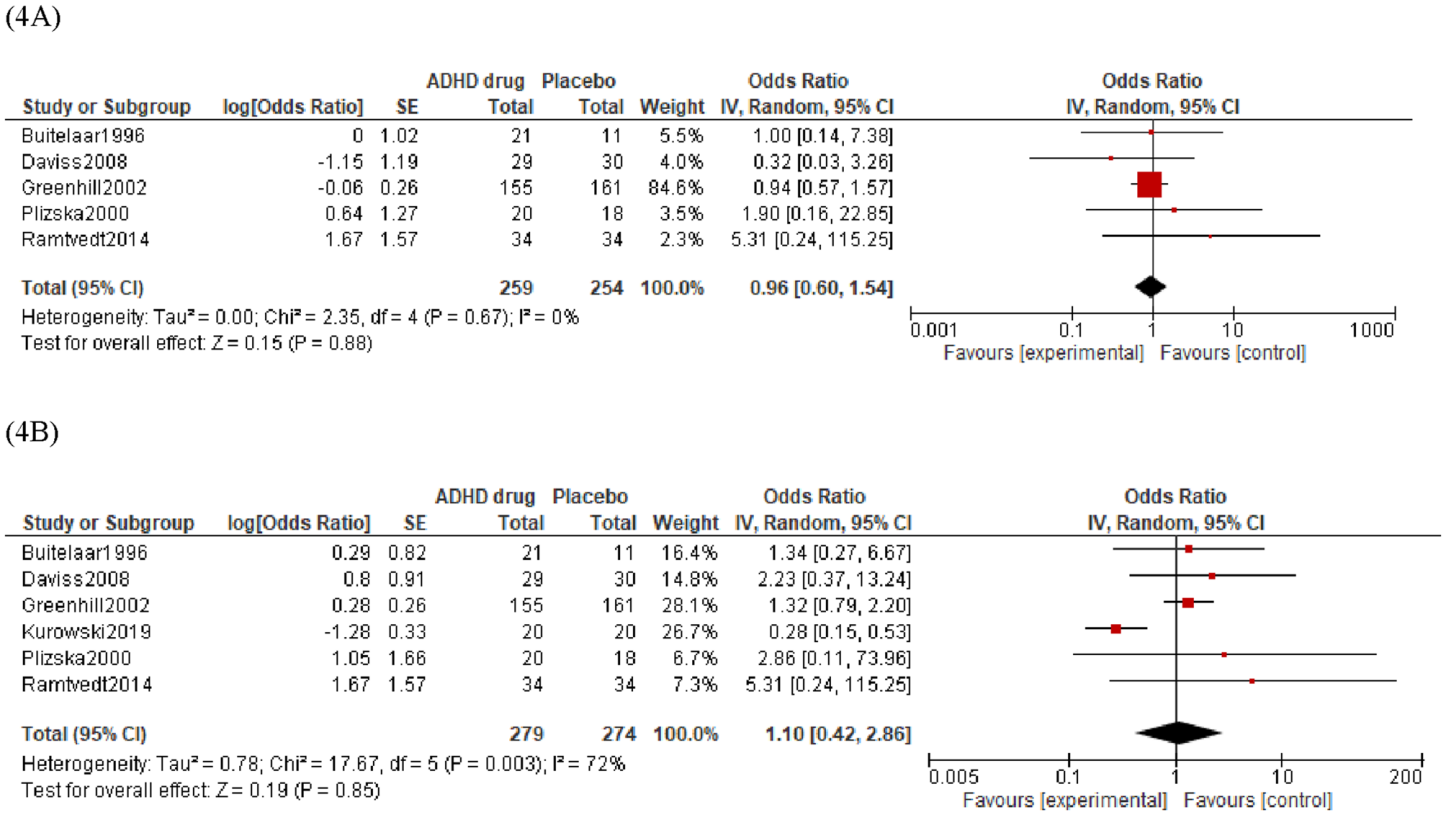
Forest plot of comparison between ADHD drug group and placebo group on Anxiety **A** and Depression **B** measured as an item on a Side Effect Rating Scale (SERS)

In the drug groups from the included trials, 17% of participants were rated as having anxiety as a side effect. In the placebo groups, 18% participants were rated as having anxiety as a side effect. Overall, there was no significant difference in the number of participants with anxiety side effects between drug and placebo groups as shown in (Fig. 4A) (OR = 0.96, 95% CI = 0.60 to 1.54, *p* = 0.67, *k* = 5). The proportion of heterogeneity effects might not be important (*I*^2^ = 0%).

In the drug groups from the included trials, 21% of participants were rated as having depression as a side effect. In the placebo groups, 15% of participants were rated as having depression as a side effect. Overall, there was no significant difference in depression side effects between drug and placebo groups as shown in (Fig. 4B) (OR = 1.10, 95% CI = 0.42 to 2.86, *p* = 0.85, *k* = 6). The proportion of heterogeneity effects was substantial (*I*^2^ = 72%). Visual inspection of the forest plot did not identify outliers.

#### Sensitivity analyses

Figures and interpretation for the sensitivity analyses are presented in Supplementary Material. Sensitivity analyses were carried out to compare whether the effects for the validated measure meta-analyses were robust to whether the data represented a change from baseline or a post-treatment score. Effect sizes and significance did not meaningfully differ when trials reporting change and post-treatment scores were meta-analysed separately for both anxiety and depression outcomes.

A sensitivity analysis was conducted including only the three trials that reported SERS item scores as a percentage of participants who had at least a moderate side effect of anxiety and/or depression (i.e., excluding trials which reported SERS item scores regardless of severity). Overall, there were no significant differences between drug and placebo groups in the presence of moderate depressive or anxious side effects.

## Discussion

The current review aimed to address the effect of taking ADHD medications compared to placebo on symptoms of anxiety and depression in RCTs with children and young people. Only 11% of eligible trials in this review reported anxiety and/or depression as an outcome or side effect, limiting the conclusions of the meta-analyses. This meta-analytic review did not yield any evidence that ADHD medication has a significant effect on anxiety or depression in children and adolescents. The absence of a significant effect was consistent when analysing trials reporting change from baseline and post-treatment scores separately, and when limiting SERS analysis to only the percentage of participants who had at least a moderate side effect of anxiety and/or depression.

These findings are consistent with Pozzi et al.’s [27] outcomes related to sadness in a meta-analysis of emotion-based AEs in ADHD medication trials, which found no difference between drug and placebo groups when analysing spontaneously reported AEs. However, they contradict Pozzi et al.’s [27] anxiety-related findings. Pozzi et al. found methylphenidate was associated with a decreased risk of treatment emergent anxiety relative to placebo. In both the current review, and a random-effects meta-analysis conducted by Coughlin and colleagues [26], no significant differences in symptoms of anxiety were found between stimulant and placebo groups. It could be argued that the outcomes reported here are more valid as they are based on rating scales that are considered a more valid measurement of child and adolescent drug trial side effects than spontaneous reports [16]. The present study included some trials also in the Coughlin and Pozzi reviews, however, the present inclusion criteria were narrower. The unique contribution of the present review comes from the inclusion criteria of ADHD being defined by clinically relevant symptoms *or* a diagnosis, and anxiety or depression being measured not by spontaneous report but by systematic measurement.

This review exemplifies that the proportion of child and adolescent drug trials of ADHD medications reporting mental health outcomes is low, and that there is substantial heterogeneity in those that do measure mental health, pointing to a clear need for widespread standardisation of mental health reporting in future ADHD drug trials. The meta-analyses conducted included just 14 trials, only 1 of which had a low risk of bias, representing only 11% of the trials deemed eligible (127) which reported analysable anxiety or depression data. Ten trials reported having measured anxiety and/or depression but did not report any data. The limited dataset included in this review was not rich enough to explore detail such as discontinuation due to mental health side effects or to compare the effects of different medication types.

Previous reviews have also been limited by the scarcity of reported mental health outcomes. Manos et al.’s (2010) literature review found only 30% of trials identified as eligible reported any EE outcomes. Of those, only 13% (6 trials) reported baseline and post treatment scores for drug and placebo groups. Similarly, Coughlin et al.’s [26] meta-analysis found that only 25% of eligible trials reported anxiety side effect data. There was marked clinical and methodological diversity in the sample of included trials and a substantial level of statistical heterogeneity in the meta-analyses of depression data. Manos et al. [25] faced a similar problem of heterogeneity in their literature review of reported EE which limited conclusions.

Collectively, current meta-analytic evidence on mental health outcomes reflects only a small portion of existing child and adolescent trials of ADHD medication. This results in low generalisability of the currently mixed findings to the wider population of children and adolescents taking medications for ADHD. This may reflect a ‘file-drawer’ problem of mental health data being omitted from trial reports or that mental health outcomes are simply not being routinely measured in these trials. There is meta-analytic evidence showing small-to-moderate effects of ADHD medication on emotion dysregulation from RCTs in adults [59]. This review also demonstrates that adult RCTs of ADHD medications measure more additional outcomes alongside core ADHD symptoms compared to child RCTs.

### Clinical implications

While the absence of an effect of ADHD medications on internalising problems across reviews of child and adolescent trials should be considered with caution due to the lack of available data, they do contrast real-world clinical anecdotal evidence. Post-licencing reporting suggests that anxiety and depression are common side effects of ADHD medications when taken by children and adolescents [20–22], which is concerning given that worldwide pharmacological treatment for ADHD is common and increasing [13, 60]. However, the available evidence here indicates that there may be no effect of ADHD medications on anxiety and depression.

### Future directions

A priority for future work is to understand shared risks for ADHD and internalising disorders, and to quantify better the effects of ADHD medications on symptoms of anxiety and depression. The key to this is widespread implementation of the standardised measurement of mental health outcomes in child and adolescent ADHD drug trials; a recommendation also made by the authors of the Manos et al. review in their companion publication on clinical practice implications [61]. 

A starting point for standardising measurement of mental health outcomes in child and adolescent ADHD drug trials could be the development of a core outcome set (COS) for ADHD. A COS is a standardised selection of outcomes that should be measured and reported for studies of a specific condition. The development of COS for health conditions improves homogeneity, clinical relevance and impartiality of clinical trial reporting and helps facilitate systematic reviewing [62]. As of April 2022, there is no established COS for ADHD in children or adults on the Core Outcome Measures in Effectiveness Trials (COMET) database [63]. Clinical trials of ADHD drugs, and meta-analytic reviews of such trials would greatly benefit from the development of a COS. The present meta-analysis demonstrates the importance of including outcome measures for depression and anxiety in a COS for clinical trials for ADHD in children and young people.

We would argue that overlooking mental health outcomes must be reconsidered by researchers, drug companies, journal reviewers and policy makers alike. Data from standardised, validated psychological rating scales of anxiety and depression symptoms in ADHD drug trials should be made available after the conclusion of every trial through online trial registries or through academic publishing. This will allow future reviews and meta-analyses to gain a valid consensus on whether ADHD medications have an impact on anxiety or depression symptoms, which will inform policy making around prescribing practices.

## Conclusion

Considering the present meta-analytic review alongside the handful of existing reviews shows that there is no evidence, thus far, from short-term randomised controlled trials that pharmacological interventions for ADHD in children and young people are associated with increased risks of anxiety or depression symptoms. However, the systemic lack of standardised measurement and reporting of mental health outcomes in such trials greatly limits the validity of current meta-analytic evidence. The disparity between evidence from short-term randomised controlled trials and real-world side effect data highlights the importance of establishing and implementing standardised, valid measurements of mental health outcomes in randomised controlled trials of ADHD medications in child and adolescent populations. Given the increased risk of internalising disorders in children and adolescents with ADHD, the increased burden of having both ADHD and an internalising disorder, and the increasing widespread prescribing of medications for ADHD, the overlooking of anxiety and depression as key outcomes of interest in child and adolescent ADHD drug trials must be reconsidered.

### Supplementary Information

Below is the link to the electronic supplementary material.

Supplementary file1 (DOCX 89 KB)

## Data Availability

Data extraction form has been made publicly available on OSF.
