# Supplementary material for: A meta-analytic review of the impact of ADHD medications on anxiety and depression in children and adolescents
Source: Eur Child Adolesc Psychiatry. 2022 May 26;32(10):1885–98. doi: 10.1007/s00787-022-02004-8 (PMC10533622; doi:10.1007/s00787-022-02004-8)
Supplement: Supplementary file 1 — Supplementary file1 (DOCX 89 KB) [file 787_2022_2004_MOESM1_ESM.docx]

**A meta-analytic review of the impact of ADHD medications on anxiety and depression in children and adolescents.**

**European Child and Adolescent Psychiatry**

**Annie Bryant**^1^, Hope Schlesinger^1^, Athina Sideri^2^, Joni Holmes^3^, Jan Buitelaar^4^ & Richard Meiser-Stedman^1^

*^1^Department of Clinical Psychology and Psychological Therapies, University of East Anglia, Norwich, UK*

*^2^Norfolk and Suffolk NHS Foundation Trust, Hellesdon Hospital, Drayton High Road, Norwich, UK*

*^3^MRC Cognition and Brain Sciences Unit, University of Cambridge, Cambridge, UK*

*^4^Radboud University, Houtlaan 4, 6525 XZ, Nijmegen, Netherlands*

Corresponding author: Annie Bryant; annie.bryant@uea.ac.uk

**Supplementary Material**

**Outcome Measures in the Included Trials**

Validated questionnaire measures of anxiety included in the meta-analyses were: parent-rated Revised Behaviour Problem Checklist (RBPC; Quay & Peterson, 1983), Anxiety/Withdrawal subscale (trial: Aman et al., 1993), parent-rated Conners Parent Rating Scale (CPRS; Conners, 1973) Anxiety subscale (trial: Brown & Sexson, 1988), parent-rated Screen for Child Anxiety Related Emotional Disorders (SCARED; Birmaher, 1997) (trial: Dell’Agnello et al., 2009), self-report Multi-Dimensional Anxiety Scale (MASC; March et al., 1997) (trial: Geller et al., 2007), self-report State and Trait Anxiety Inventory (STAI, Spielberger et al., 1983) State Anxiety scale and, for children aged under 14 years, State and Trait Anxiety Inventory for Children (STAI-C, Spielberger et al., 1973) (trial: Griffiths et al., 2018), and clinician-rated Conners Comprehensive Behaviour Rating Scales (CBRS; Conners, 2008) Generalised Anxiety Disorder (GAD) subscale (trial: Lin et al., 2014).

Validated questionnaire measures of depression included in the meta-analyses were: clinician-rated Children’s Depression Rating Scale-Revised (CDRS-R, Poznanski & Mokros, 1996) (trials: Bangs et al., 2007; Dell’Agnello et al., 2009; Michelson et al., 2001), self-report Depression, Anxiety and Stress Scale (DASS, Lovibond & Lovibond, 1996) (trial: Griffiths et al., 2018), and clinician-rated Conners Comprehensive Behaviour Rating Scales (CBRS; Conners, 2008) Major Depressive Disorder (MDD) subscale (trial: Lin et al., 2014).

SERS measures of anxiety and depression items included in the meta-analyses were: Barkley Stimulant Side Effect Rating Scale (BSSERS; Barkley, 1990) (trials: Buitelaar et al., 1996; Ramtvedt et al., 2014), Pittsburgh Side Effect Rating Scale (PSERS; Pelham, 1993) (trials: Daviss et al., 2008; Greenhill et al., 2002; Kurowski et al., 2019), and Multi-Modality Treatment of ADHD side effects scale (Greenhill et al., 1996) (trial: Pliszka et al., 2000). All SERS measures were parent-rated. In the PROSPERO registration for this review, only the BSSERS and PSERS were mentioned as SERS outcomes of interest. However, during the systematic review the Multi-Modality Treatment of ADHD side effects scale was found to be similar to these measures and available information about the measure showed it was suitable for inclusion in the meta-analysis.

**References for Outcome Measures**

Barkley RA (1990) Attention-deficit hyperactivity disorder: A handbook for diagnosis and treatment. Guildford Press.

Birmaher B (1997) The Screen for Child Anxiety-Related Emotional Disorders (SCARED): Scale construction and psychometric characteristics*.* J Am Acad Child Psy 36*:*545–553.

Conners CK (1973) Rating scales for use in drug studies with children. Psychopharmacology Bulletin 24–29.

Conners CK (2008) Conners Comprehensive Behavior Rating Scales: Manual. Multi-Health Systems.

Greenhill LL, Abikoff HB, Arnold LE et al. (1996). Medication treatment strategies in the MTA study: Relevance to clinicians and researchers. J Am Acad Child Psy 35*:*1304–1313. <https://doi.org/10.1097/00004583-199610000-00017>

Lovibond SH, Lovibond PF (1996) Manual for the Depression, Anxiety, Stress Scales. Psychology Foundation of Australia.

March JS, Parker JDA, Sullivan K, Stallings P, Conners CK (1997) The Multidimensional Anxiety Scale for Children (MASC): Factor structure, reliability, and validity. J Am Acad Child Psy 36*:*554–565. <https://doi.org/10.1097/00004583-199704000-00019>

Pelham WE (1993) Pharmacotherapy for children with attention-deficit hyperactivity disorder. *School* Psychology Rev 22:199–227. <https://doi.org/10.1080/02796015.1993.12085647>

Poznanski E, Mokros H (1996) Children’s Depression Rating Scale, Revised (CDRS-R). WPS.

Quay HC, Peterson DR (1983) Interim manual for the Revised Behaviour Problem Checklist (1st edition). University of Miami.

Spielberger CD, Edwards RE, Lushene J, Montouri D (1973) Preliminary test manual for the State-Trait Anxiety Inventory for Children. Pers Indiv Differ 2. <https://doi.org/10.1016/0191-8869(81)90005-2>

**Supplementary Figure 1**

*Forest Plot of Comparison Between ADHD Drug Group and Placebo Group on Anxiety Change Scores as Measured by Validated Questionnaires*


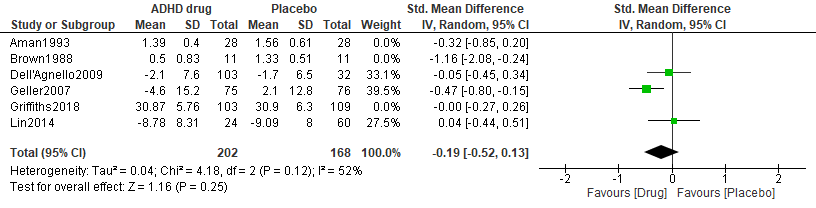


For anxiety change scores measured by validated questionnaires, the magnitude of the effect is small and favours ADHD drugs over placebo (SMD= -0.19, 95% CI= -.52-.13, *p*= 0.12, *n*= 370, *k*= 3). The proportion of heterogeneity effects were modest (I^2^= 52%).

**Supplementary Figure 2**

*Forest Plot of Comparison Between ADHD Drug Group and Placebo Group on Anxiety Post-Treatment Scores as Measured by Validated Questionnaires*


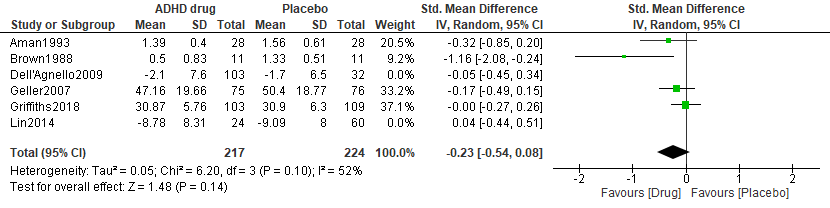


For anxiety post-treatment scores measured by validated questionnaires, the magnitude of the effect is small and favours ADHD drugs over placebo (SMD= -0.23, 95% CI= -.54-.08, *p*= 0.10, *n*= 441, *k*= 4). The proportion of heterogeneity effects were modest (I^2^= 52%).

**Supplementary Figure 3**

*Forest Plot of Comparison Between ADHD Drug Group and Placebo Group on Depression Change Scores as Measured by Validated Questionnaires*


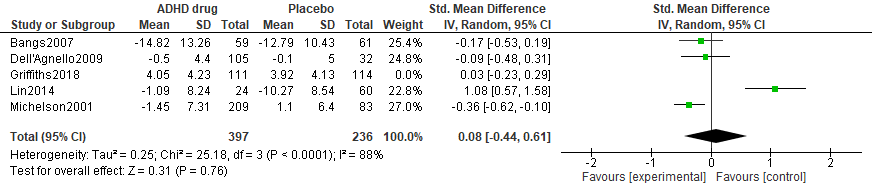


For depression change from baseline scores measured by validated questionnaires, the magnitude of the effect is small and favours placebo over ADHD drugs (SMD= 0.08, 95% CI= -.44-.61, *p*= <.001, *n*= 633, *k*= 4). The proportion of heterogeneity effects was substantial (I^2^= 88%). As only one trial reported post-treatment scores for depression, a separate meta-analysis for these data was not appropriate.

**Supplementary Figure 4**

*Forest Plot of Comparison Between ADHD Drug Group and Placebo Group on At Least Moderate Anxiety Measured on a Side Effect Rating Scale (SERS)*


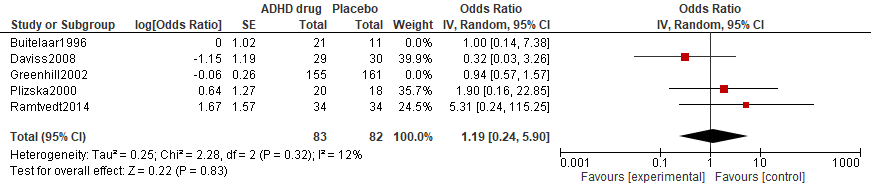


Overall, there was no significant difference in anxiety side effects between drug and placebo groups (OR= 1.19, 95% CI= .24-5.90, p= 0.32, k= 3). The proportion of heterogeneity effects might not be important (I^2^= 12%).

**Supplementary Figure 5**

*Forest Plot of Comparison Between ADHD Drug Group and Placebo Group on At Least Moderate Depression Measured on a Side Effect Rating Scale (SERS)*


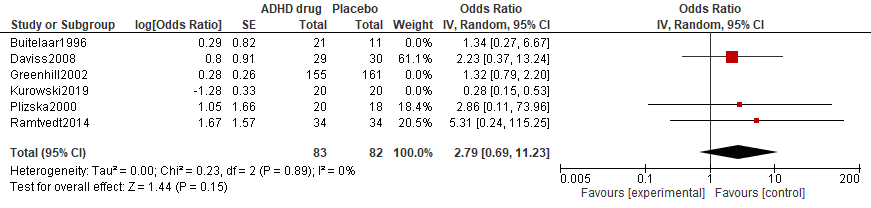


Overall, there was no significant difference in depression side effects between drug and placebo groups (OR= 2.79, 95% CI= .69-11.23, p= 0.89, k= 3). The proportion of heterogeneity effects might not be important (I^2^= 0%).

**Supplementary Table 1**

PRISMA Checklist

| **Section/topic** | **#** | **Checklist item** | **Reported on page #** |
| --- | --- | --- | --- |
| **TITLE** | | |  |
| Title | 1 | Identify the report as a systematic review, meta-analysis, or both. | 1 |
| **ABSTRACT** | | |  |
| Structured summary | 2 | Provide a structured summary including, as applicable: background; objectives; data sources; study eligibility criteria, participants, and interventions; study appraisal and synthesis methods; results; limitations; conclusions and implications of key findings; systematic review registration number. | 2 |
| **INTRODUCTION** | | |  |
| Rationale | 3 | Describe the rationale for the review in the context of what is already known. | 4, 5 |
| Objectives | 4 | Provide an explicit statement of questions being addressed with reference to participants, interventions, comparisons, outcomes, and study design (PICOS). | 5, 6 |
| **METHODS** | | |  |
| Protocol and registration | 5 | Indicate if a review protocol exists, if and where it can be accessed (e.g., Web address), and, if available, provide registration information including registration number. | 5 |
| Eligibility criteria | 6 | Specify study characteristics (e.g., PICOS, length of follow-up) and report characteristics (e.g., years considered, language, publication status) used as criteria for eligibility, giving rationale. | 5, 6 |
| Information sources | 7 | Describe all information sources (e.g., databases with dates of coverage, contact with study authors to identify additional studies) in the search and date last searched. | 5 |
| Search | 8 | Present full electronic search strategy for at least one database, including any limits used, such that it could be repeated. | 5, 6 |
| Study selection | 9 | State the process for selecting studies (i.e., screening, eligibility, included in systematic review, and, if applicable, included in the meta-analysis). | 6, 7 |
| Data collection process | 10 | Describe method of data extraction from reports (e.g., piloted forms, independently, in duplicate) and any processes for obtaining and confirming data from investigators. | 7, 8, 9 |
| Data items | 11 | List and define all variables for which data were sought (e.g., PICOS, funding sources) and any assumptions and simplifications made. | 7, 8, 9 |
| Risk of bias in individual studies | 12 | Describe methods used for assessing risk of bias of individual studies (including specification of whether this was done at the study or outcome level), and how this information is to be used in any data synthesis. | 8 |
| Summary measures | 13 | State the principal summary measures (e.g., risk ratio, difference in means). | 7, 8 |
| Synthesis of results | 14 | Describe the methods of handling data and combining results of studies, if done, including measures of consistency (e.g., I^2^) for each meta-analysis. | 7, 8 |

Page 1 of 2

| **Section/topic** | **#** | **Checklist item** | **Reported on page #** |
| --- | --- | --- | --- |
| Risk of bias across studies | 15 | Specify any assessment of risk of bias that may affect the cumulative evidence (e.g., publication bias, selective reporting within studies). | 8 |
| Additional analyses | 16 | Describe methods of additional analyses (e.g., sensitivity or subgroup analyses, meta-regression), if done, indicating which were pre-specified. | 9 |
| **RESULTS** | | |  |
| Study selection | 17 | Give numbers of studies screened, assessed for eligibility, and included in the review, with reasons for exclusions at each stage, ideally with a flow diagram. | 9, 24 |
| Study characteristics | 18 | For each study, present characteristics for which data were extracted (e.g., study size, PICOS, follow-up period) and provide the citations. | 9, 10, 22, 23 |
| Risk of bias within studies | 19 | Present data on risk of bias of each study and, if available, any outcome level assessment (see item 12). | 10, 25 |
| Results of individual studies | 20 | For all outcomes considered (benefits or harms), present, for each study: (a) simple summary data for each intervention group (b) effect estimates and confidence intervals, ideally with a forest plot. | 10, 11, 26, 27 |
| Synthesis of results | 21 | Present results of each meta-analysis done, including confidence intervals and measures of consistency. | 10, 11, 26, 27 |
| Risk of bias across studies | 22 | Present results of any assessment of risk of bias across studies (see Item 15). | 10, 25 |
| Additional analysis | 23 | Give results of additional analyses, if done (e.g., sensitivity or subgroup analyses, meta-regression [see Item 16]). | 11 [Supplementary File] |
| **DISCUSSION** | | |  |
| Summary of evidence | 24 | Summarize the main findings including the strength of evidence for each main outcome; consider their relevance to key groups (e.g., healthcare providers, users, and policy makers). | 11, 12, 13 |
| Limitations | 25 | Discuss limitations at study and outcome level (e.g., risk of bias), and at review-level (e.g., incomplete retrieval of identified research, reporting bias). | 12 |
| Conclusions | 26 | Provide a general interpretation of the results in the context of other evidence, and implications for future research. | 14 |
| **FUNDING** | | |  |
| Funding | 27 | Describe sources of funding for the systematic review and other support (e.g., supply of data); role of funders for the systematic review. | 1 |

*From:*  Moher D, Liberati A, Tetzlaff J, Altman DG, The PRISMA Group (2009). Preferred Reporting Items for Systematic Reviews and Meta-Analyses: The PRISMA Statement. PLoS Med 6(7): e1000097. doi:10.1371/journal.pmed1000097

For more information, visit: **www.prisma-statement.org**.

Page 2 of 2
